# User, expert, and construct validation of a new colonoscopy simulator: correlation with National Endoscopy Database key performance indicators

**DOI:** 10.1016/j.igie.2025.08.007

**Published:** 2025-08-22

**Authors:** Olaolu Olabintan, Adeyeye Ademola, Mabel Tanne, Homira Ayubi, Ali Eqbal, Mehul Patel, Andrew Emmanuel, Shraddha Gulati, Amyn Haji, Bu’Hussain Hayee

**Affiliations:** 1 Department of Endoscopy, King's College Hospital, London, United Kingdom; 2 Department of Gastroenterology, King's College Hospital, London, United Kingdom; 3 Department of Colorectal Surgery, King's College Hospital, London, United Kingdom; 4 Faculty of Life Sciences & Medicine, King's College London, London, United Kingdom

## Abstract

**Background and Aims:**

Training in flexible gastrointestinal endoscopy is increasingly challenging. Simulation tools may help impart essential skills, but existing simulators lack real-time feedback, may be unrealistic, and are not aligned with recognized key performance indicators (KPIs). As a result, translating from simulator to real-world procedures has achieved variable outcomes. The Mikoto colonoscopy simulator (R Zero Inc, Tokyo, Japan) aims to address this gap by providing real-time feedback based on procedural dynamics, including patient comfort, producing a single performance-focused score (out of a maximum possible 100; the Mikoto Simulator Score [MSS]).

We sought to establish construct and user validation for the MSS, with endoscopists' KPIs and structured user feedback.

**Methods:**

Twenty endoscopists of varying experience levels were recruited and categorized into novice, training, competent, and expert experience levels, based on lifetime colonoscopy numbers (national accreditation criteria). Participants provided their United Kingdom National Endoscopy Database (NED) KPIs before using the simulator. After standardized introduction and acclimatization, each then performed 3 full colonoscopies on the simulator, with the main test parameters being cecal intubation time and MSS. User validity was determined by means of a structured feedback questionnaire assessing utility and realism.

**Results:**

Significant differences were observed in median NED KPIs and MSS across all experience levels (n = 5 in each group, *P* = .046), with a linear correlation between lifetime colonoscopy numbers and MSS (*P* = .027). There were also highly significant correlations demonstrated between MSS and NED colonoscopy comfort score (*P* < .001), polyp detection rate (*P* < .001), and cecal intubation rate (*P* < .001).

**Conclusions:**

The Mikoto simulator demonstrates close alignment with NED KPIs for colonoscopy, with linear correlation in most cases, providing initial validation as an indicator of endoscopic competence in a nonpatient-contact setting. Further studies are warranted to assess integration into endoscopy training. The Mikoto simulator represents a promising tool for enhancing endoscopic training and improving patient outcomes.

## Introduction

Proficiency in gastrointestinal (GI) endoscopy is essential for a substantial number of diagnostic and therapeutic interventions in gastroenterology and upper and lower GI surgery.^1^ The acquisition and refinement of endoscopic skills represents a steep learning curve (for colonoscopy in this context), particularly during early stages of training.^2^ Simulation-based training has emerged as a valuable tool for bridging this gap, offering a safe and controlled environment for trainees to practice essential maneuvers and procedures.^3^ Use of simulation in structured training has demonstrated some benefits within the simulation environment,^1^^,^^3^ but there is some doubt as to whether this translates into “real-world” clinical utility.^4^ This may be because current endoscopy simulators lack the realism of haptic feedback^3^ and have not historically been validated for key performance indicators (KPIs), including cecal intubation rate (CIR), polyp detection rate (PDR), and colonoscopy comfort score (CCS). These data are routinely collected and reported in the United Kingdom National Endoscopy Database (NED) initiative (https://nedpilot.thejag.org.uk/).^5^ In the United Kingdom, colonoscopy training is highly regulated, with trainees providing real-time data to assess progress via the Joint Advisory Group endoscopy training system (https://jets.thejag.org.uk/).^5^ Assessment of technical and nontechnical skills occurs using the Direct Observation of Procedural Skills tool at the start, during, and end of each hospital rotation (12-month attachments). Trainee colonoscopists are deemed competent, and therefore independent, when they achieve target KPIs, upon the completion of a minimum of 280 procedures.

The Mikoto (R Zero Inc, Tokyo, Japan) colonoscopy simulator has been developed to closely replicate real-life endoscopy, with a particular emphasis on accurate visual and haptic feedback. Furthermore, the associated central processing unit monitors performance to generate a comprehensive score based on factors such as procedural comfort, mucosal exposure, and successful navigation to key anatomical landmarks within the colon. This gives Mikoto the potential to be used as a clinically relevant training tool for colonoscopy accreditation.^6^ We sought to establish construct and user validation for this novel colonoscopy simulator, as well as to determine whether performance on the simulator could be correlated with NED KPIs.

## Methods

### Simulator

**Figure 1:**
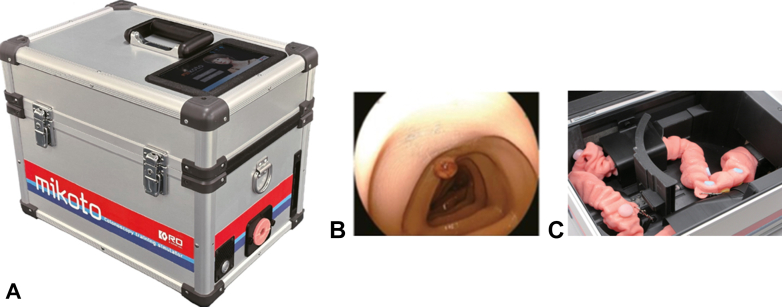
The Mikoto colonoscopy training simulator box (**A**). Live luminal view of the Mikoto colonoscopy simulator when in use (**B**). Silicon resin colon model inside the simulator box (**C**).

The Mikoto (Fig. 1) is a humanoid-robot simulator designed to enable endoscopists to practice scope insertion in a manner similar to performing a real colonoscopy. The device is constructed from a thin, flexible silicon resin, making it more adaptable than other colon simulators. Its 3-dimensional model was developed using computed tomography imaging data of the human colon.^7^ With the use of real-time physical data, the central processing unit generates a composite score (maximum 100) at procedure end. This score is determined by (1) time to reach the cecum, (2) loop formation, (3) application of abdominal pressure, and (4) arrival to the sigmoid-descending junction and appendicular orifice. The option of simulated use of sedation can be selected by the endoscopist, and pre-determined configurations of the colon and fixations can be altered to increase procedural challenge (Easy, Standard, Advance, and Advance-2). The simulator was provided on loan to our institution by Fujifilm (Tokyo, Japan) for the duration of the study; however, the company had no involvement in the study design, data collection, or data analysis.

### Participants

Twenty endoscopists of varying experience levels were recruited to participate in the study. Participants were categorized into 4 groups on the basis of their lifetime colonoscopy (LC) numbers, which were recorded in the NED: novice (<50), training (50-280), competent (281-2000), and expert (>2000). These categories were selected on the basis of several comparable studies^1^ as well as the threshold of 280 lifetime colonoscopies as the minimum required by the United Kingdom training system for an endoscopist to be deemed competent to perform independent colonoscopy.^6^ With a requirement of 1000 lifetime colonoscopies to be recognized as a bowel cancer screening endoscopist in the United Kingdom, we opted to set a greater standard for our expert category, broadly in line with other studies.^8^

Three NED KPI data—CIR (%), PDR (%), and CCS—also were obtained for each participant, just before study start. The CCS is a 4-point Likert scale, reported by endoscopy nurses after each procedure, on the basis of patient's experience and often influenced by the loop formation during the procedure, extracted by the NED software interface from each hospital's endoscopy reporting system and transformed to a score ranging from 0 to 100, with higher score indicating greater patient discomfort.^5^

### Intervention

To standardize conditions, all participants used the same endoscope, the Fujifilm EC-760S-G, equipped with ColoAssist (Fujifilm Corporation), a 3-dimensional imaging and navigation system offering real-time feedback on scope position and loop formation. The ColoAssist was also switched on for all participants to maintain consistency across all procedures. Endoscopists unfamiliar with the colonoscope model were offered a 10-minute practice session on the Colonoscopy Simulator Type II model (Adam, Rouilly Ltd, Sittingbourne, United Kingdom) as a commonly used latex training model in the United Kingdom, before attempting the Mikoto simulator.

After acclimatization, each participant completed 3 full colonoscopies on the Mikoto simulator, using the “standard” challenge setting, with no interruptions between attempts, and with the “colo-assist.” Cecal intubation time (CIT) and Mikoto scores were recorded after each attempt, and the best score was recorded. None of the participants had previous experience using the simulator or were involved in its development.

### Questionnaire

**Supplementary Figure 1:**
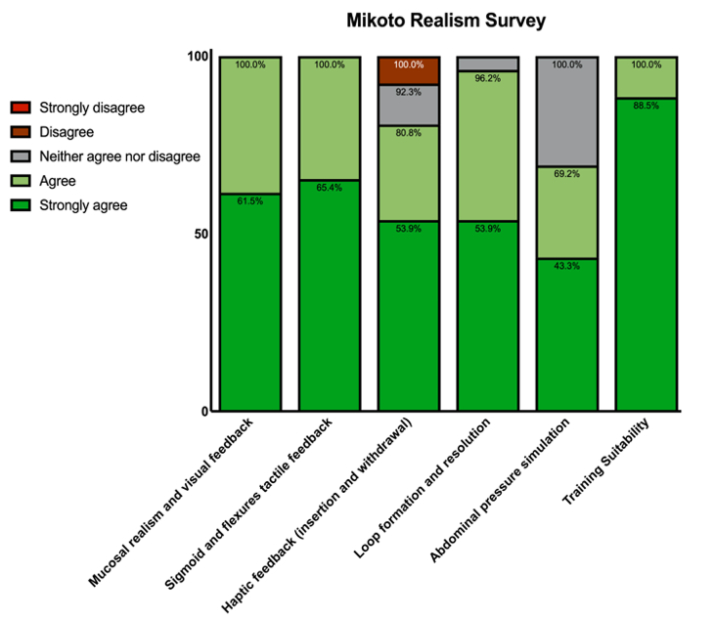
Mikoto Realism Survey summary results for all participants.

After the completion of 3 attempts on the Mikoto simulator, all endoscopists completed a questionnaire to objectively assess the realism of the simulator. The questionnaire focused on 3 domains: (1) realism of imaging, (2) control and haptic feedback, and (3) training suitability. Responses were expressed on a 5-point Likert scale varying from 1 (strongly agree) to 5 (strongly disagree). The results presented primarily focus on feedback from competent and expert endoscopists, as their insights are most relevant for qualitative evaluation (n = 10) (Supplementary Fig. 1, available online at www.igiejournal.org for survey results from all participants).

### Outcome measures and sample size calculation

The primary outcome measure was the Mikoto Simulator Score (MSS) between the different groups stratified by experience level (lifetime procedure count). Secondary outcome measures included correlation between MSS and LC number, between 2 Mikoto read-outs (MSS and Mikoto cecal intubation time [M-CIT]) and NED KPIs (CIR, PDR, and CCS), and variation of these across the different experience levels.

The sample size calculation was based on a 4:1 difference in the CIT between the competent group and novice group. This estimation was informed by findings from a similar study that compared the “time to finish” on a task between novice and “experienced” groups using a colon simulator.^1^ A total sample size of 20 participants is required to detect a statistically significant difference, with an alpha level set at 0.05 and a beta level of 0.2, thereby achieving a statistical power of 80%.

### Statistical analysis

Descriptive statistics were used to summaries participant demographics and performance scores, with mean (standard deviation) or median (interquartile range) for contiguous and noncontiguous variables, respectively. Linear regression analysis was used to measure relationship between 2 variables. Quantitative variables were analyzed using the *t* test, whereas qualitative variables were analyzed using the χ^2^ test or Fisher exact test. A 1-way analysis of variance was conducted as appropriate. A *P* value <.05 was considered statistically significant.

## Results

### Mikoto simulator activity data

**Figure 2:**
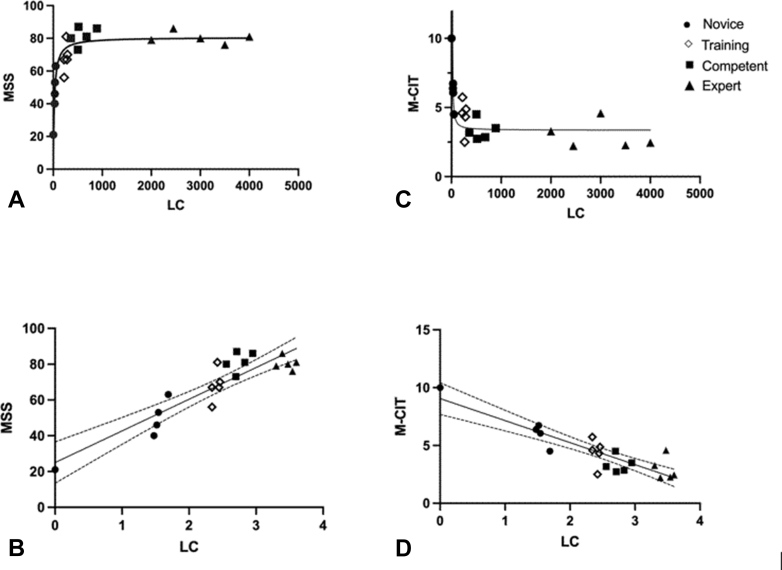
Relationship between lifetime colonoscopy (LC) number, Mikoto Simulator Score (MSS), and Mikoto cecal intubation time (M-CIT). **A,** Raw plot between MSS and LC; (**B**) linear regression between MSS and Log 10 of LC; (**C**) raw plot between M-CIT and LC; (**D**) linear regression between M-CIT and Log 10 of LC.

The median (range) LC numbers of the novice, training, competent, and expert groups were 33 (1-49), 265 (220-289), 515 (380-890), and 3000 (2000-4000), respectively, and the NED KPIs, MSS, and M-CIT were significantly different across the 4 experience levels (Table 1). There was a statistically significant, linear correlation observed between MSS and LC (*P* = .026) and M-CIT and LC (*P* = .0176) (Fig. 2). It is, however, noted that this relationship begins to plateau. Using the GraphPad Prism software (version 10.4.1; GraphPad Software, San Diego, Calif, USA), we were able to intrapolate the values at this plateau point to be equivalent to 360 lifetime colonoscopies, which correlate with the MSS of 81 and M-CIT of 3 minutes, 21 seconds.

**Table 1:** Relationship between LC, NED KPIs, MSS, and M-CIT across all 4 experience levels

	Novice (n = 5)	Training (n = 5)	Competent (n = 5)	Expert (n = 5)	*P* value
LC	33 (1-49)	265 (220-280)	515 (380-890)	3000 (2000-4000)	<.001
PDR, %	0 (0-8)	20 (10-26)	27 (9-31.9)	45 (40-55)	.003
CIR, %	37 (0-68)	92 (60-95)	97 (96-98)	99 (99-99)	.005
CCS, %	40 (35-80)	15 (8-40)	4 (2-5)	0.8 (0.2-1.5)	.004
MSS	46 (21-63)	67 (56-81)	81 (63-87)	80 (76-86)	.005
M-CIT, minutes	6:04 (4:30-10:00)	4:36 (2:32-5:44)	3:11 (2:44-4:30)	2:27 (2:13-4:35)	.003

Data presented as median (interquartile range) (LC, MSS, and M-CIT).

*CCS*, Colonoscopy Comfort Score; *CIR*, cecal intubation rate; *KPI*, key performance indicator; *LC*, lifetime colonoscopy; *M-CIT*, Mikoto cecal intubation time; *MSS*, Mikoto Simulator Score; *NED*, National Endoscopy Database; *PDR*, polyp detection rate.

**Figure 3:**
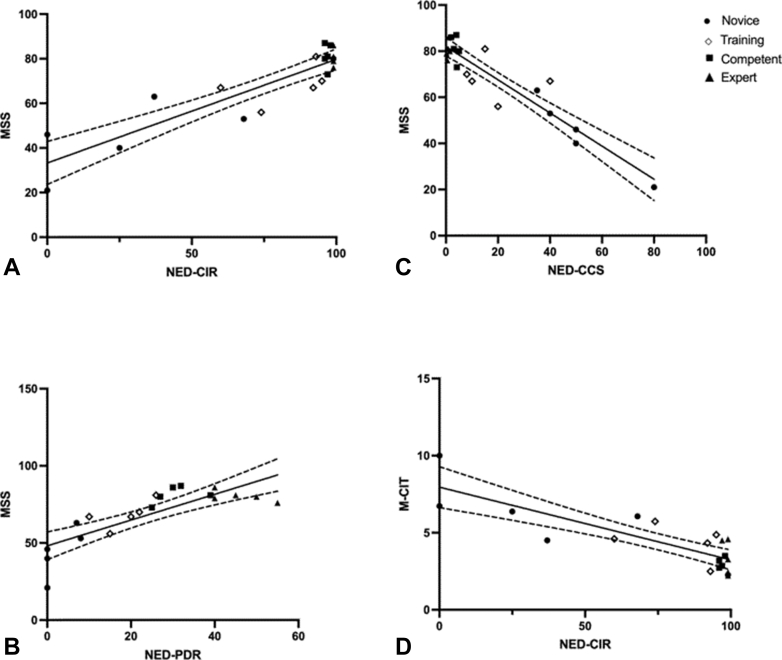
Correlation between National Endoscopy Database key performance indicators, Mikoto Simulator Score (MSS), and Mikoto–cecal intubation time (M-CIT). **A,** Correlation between NED–cecal intubation rate (NED-CIR) and MSS; (**B**) correlation between NED–polyp detection rate (NED-PDR) and MSS; (**C**) correlation between NED–Colonoscopy Comfort Score (NED-CCS) and MSS; (**D**) correlation between NED–cecal intubation rate (NED-CIR) and M-CIT.

There was a statistically significant correlation between MSS and NED-CCS (*P* < .001), NED-PDR (*P* < .001), and NED-CIR (*P* < .001) individually (Fig. 3). There was also a significant correlation between M-CIT and NED-CIR (*P* < .001) (Fig. 3). Finally, analysis of MSS and the 4 experience levels demonstrated a strong statistical significance (*P* < .001, analysis of variance) and between novice versus training (*P* = .0067), novice versus competent (*P* < .001), and novice versus expert (*P* < .001). There was no statistical difference observed between competent versus expert (*P* = .998) and training versus competent (*P* = .175).

**Figure 4:**
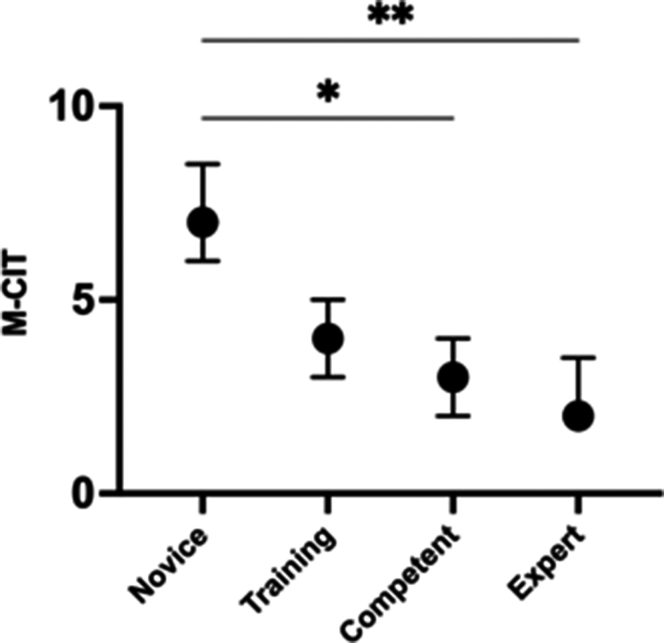
Analysis of variance results for Mikoto cecal intubation time (M-CIT) across 4 experience levels. Data are presented as median with interquartile range. Post hoc analysis revealed statistically significant differences: ∗*P* < .05 between novice and competent, and ∗∗*P* < .001 between novice and expert.

Across all 4 experience levels, there was a statistically significant difference in the M-CIT (*P* = .004, Kruskal-Wallis test) (Fig. 4; Supplementary Material, available online at www.igiejournal.org). Further analysis demonstrated a statistical difference between the novice versus competent (*P* < .021) and novice versus expert (*P* = .005), but there was no statistical difference shown between the competent versus expert group (*P* = 1), training versus competent (*P* = 1), and training versus expert (*P* = .983).

### Realism survey: expert validation

**Figure 5:**
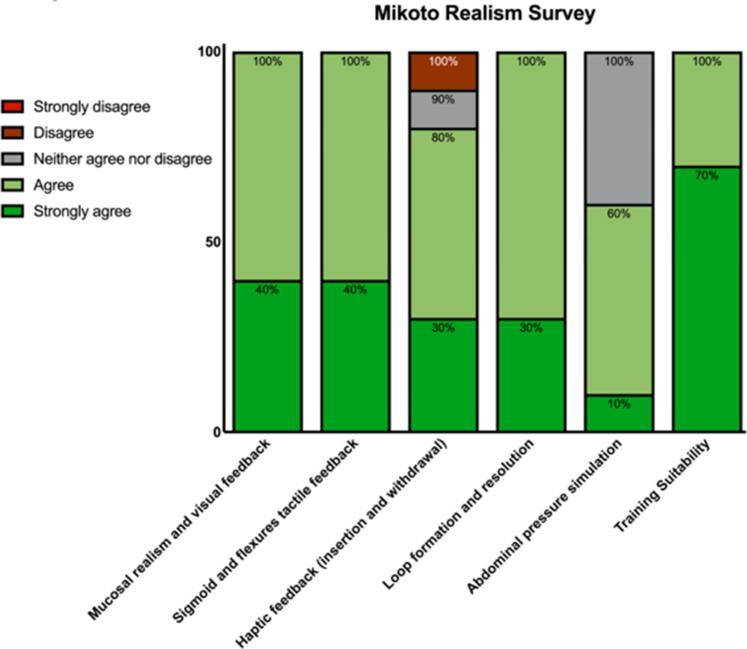
Mikoto Realism Survey summary result (competent and expert).

All respondents agreed or strongly agreed that the visual feedback provided by the Mikoto simulator closely resembled a real colonoscopy procedure and that the endoscope control accurately reflected in vivo handling (Fig. 5). In addition, 80% of respondents agreed or strongly agreed that the haptic feedback was realistic, whereas 10% disagreed and 10% were neutral. All respondents agreed or strongly agreed that the simulator accurately replicates loop formation and response to loop resolution techniques, although 40% were neutral regarding the realism of the abdominal pressure feedback. Finally, all the respondents agreed that this simulator is a suitable training tool for novice endoscopists.

## Discussion

To our knowledge, this is the first study to evaluate the comparability of real-time performance scores from any colonoscopy simulator with recognized KPIs. Our findings indicate a linear correlation between Mikoto read-outs (MSS and M-CIT) and LC numbers. In addition, the MSS appears to differentiate novice and competent endoscopists. Taken together, these analyses suggests that the Mikoto colon simulator could be used as an indicator of early colonoscopy competence. In our cohort, the MSS was not able to differentiate between competent and expert colonoscopists, with the greatest score of 87 and 86, respectively. This is broadly in line with the findings of other studies, where the value of simulator training appears to be in the novice or early training cohort.^4^^,^^9^ This may merely reflect that the skills acquired on this simulator (scope handling, tip control, and loop resolution) are skills that have been established through competency and pre-existing training. The simulator may thus be less beneficial for competent endoscopists, but further work is required before concluding this (including sampling a larger cohort), particularly to determine whether there may be an “upskilling” effect in this cohort when exposed to focused simulator training.

Although the MSS correlated with LC and NED KPIs, the “plateauing” of this effect at around 360 lifetime colonoscopies is an important finding. This is broadly in line with the current United Kingdom recommendations for a minimum lifetime experience number of 280 colonoscopies (assuming target KPIs are met). Although further work would be required, these data suggest that simulator training with Mikoto read-outs could be used as a threshold indicator of early competence before (or during) training in real-world procedures.

A strong correlation established between MSS versus NED-PDR and M-CIT versus NED-CIR across all 4 experience levels suggest the Mikoto simulator effectively captures other real-life procedural metrics indicative of endoscopic competence. This is particularly reassuring, given the established importance of adenoma detection rate and CIR as a universal quality measure in colonoscopy.^6^

Although there are a few studies in which a colon simulator has been able to show a correlation with comfort scores, this is not often reported.^1^^,^^3^^,^^4^^,^^9^ The overall MSS captures colon elongation, loop formation, and loop resolution, which are important metrics responsible for overall comfort during colonoscopy. Our study has been able to show a strong correlation between the MSS and NED-CCS, thus indicating potential value in training where the eventual aim is to improve patient comfort during colonoscopy.

User and expert validation from other colon simulators have shown mixed results with regard to haptic feedback.^9^ An expert validation from Koch et al^1^ reports the haptic feedback of their simulator to be “doubtful,” with a score of 2.57 out of 4, and anatomical representation rated 2.58. The Mikoto simulator demonstrates a realistic representation of visual, tactile, and haptic feedback, with survey respondents also unanimously agreeing that it is a good endoscopy training tool.

Although this study was conducted at a single tertiary center, which potentially limits the generalizability of the findings, we included a sufficiently diverse sample of endoscopists at various experience levels, consistent with the scope of prior comparable studies. A limitation of the simulation model is the use of surrogate markers such as loop formation and colonic distension to infer patient discomfort. Although this is clinically relevant, it does not fully reflect the range of patient responses observed during real live procedures.

In conclusion, our study demonstrates that the MSS aligns closely with established NED KPIs for colonoscopy, in all comparisons across nearly all experience levels, suggesting its potential as a colonoscopy training and assessment tool. This could be incorporated in formal colonoscopy training, especially for endoscopists in the very early stage of their training. Given the current challenges in colonoscopy training, and the need for effective skills development, the Mikoto colon simulator represents a promising tool for enhancing training and improving the quality of gastrointestinal endoscopy. Future research should focus on prospective studies in a larger training cohort, as well as in the competent cohort specifically. The use of such a realistic validated simulator may “front-load” the acquisition of skills associated with colonoscopy competence. Integration into endoscopy training programs could potentially reduce the time required to achieve diagnostic colonoscopy competence; however, further evaluation through a large, multicenter study is needed to confirm these findings.

## Patient Consent

There were no patients involved in this study. This study was with a humanoid robotic colonoscopy simulator and clinicians.

## Disclosure

All authors disclosed no financial relationships.

## Appendix

Mikoto Kruskal -Wallis Test for MCIT across all competent levels
